# Challenges in Treating Pediatric Cancer Patients during the COVID-19 Pandemic: Balancing Risks and Care

**DOI:** 10.3390/v16050690

**Published:** 2024-04-26

**Authors:** Juan Luis Chávez-Pacheco, Manuel Castillejos-López, Laura M. Hernández-Regino, Liliana Velasco-Hidalgo, Marta Zapata-Tarres, Valeria Correa-Carranza, Guillermo Rosario-Méndez, Rehotbevely Barrientos-Ríos, Arnoldo Aquino-Gálvez, Luz María Torres-Espíndola

**Affiliations:** 1 Pharmacology Laboratory, National Institute of Pediatrics, Mexico City 04530, Mexico; jchavez_pacheco@hotmail.com (J.L.C.-P.); lauris26@gmail.com (L.M.H.-R.); valcorcar@gmail.com (V.C.-C.); guillermo.rosario403@gmail.com (G.R.-M.); 2 Epidemiology and Infectology, National Institute of Respiratory Diseases, Mexico City 14080, Mexico; mcastillejos@gmail.com; 3 Oncology Service, National Institute of Pediatrics, Mexico City 04530, Mexico; lilianav@hotmail.com; 4 Head of Research Coordination at Mexican Social Security Institute Foundation, Mexico City 06600, Mexico; mzapatatarres@gmail.com; 5 Cytogenetics Laboratory, Department of Human Genetics, National Institute of Pediatrics, Mexico City 04530, Mexico; rehotbevely@gmail.com; 6 Molecular Biology Laboratory, Pulmonary Fibrosis Department, National Institute of Respiratory Diseases, Mexico City 14080, Mexico

**Keywords:** COVID-19, treatment, SARS-CoV-2, pediatric cancer

## Abstract

The COVID-19 pandemic has resulted in millions of fatalities worldwide. The case of pediatric cancer patients stands out since, despite being considered a population at risk, few studies have been carried out concerning symptom detection or the description of the mechanisms capable of modifying the course of the COVID-19 disease, such as the interaction and response between the virus and the treatment given to cancer patients. By synthesizing existing studies, this paper aims to expose the treatment challenges for pediatric patients with COVID-19 in an oncology context. Additionally, this updated review includes studies that utilized the antiviral agents Remdesivir and Paxlovid^TM^ in pediatric cancer patients. There is no specific treatment designed exclusively for pediatric cancer patients dealing with COVID-19, and it is advisable to avoid self-medication to prevent potential side effects. Managing COVID-19 in pediatric cancer patients is indeed a substantial challenge. New strategies, such as chemotherapy application rooms, have been implemented for children with cancer who were positive for COVID-19 but asymptomatic since the risk of disease progression is greater than the risk of complications from SARS-CoV-2.

## 1. Introduction

Coronaviruses (CoVs) are a part of the Coronaviridae family and the Nidovirales order, specifically, the Betacoronavirus genus, which can potentially cause mammalian illness [1]. SARS-CoV-2 is a type of beta coronavirus with positive single-stranded RNA and notable mutational and recombination capabilities. It possesses unique components, such as the spike protein (S), to facilitate its replication [2].

In December 2019, a novel coronavirus was discovered in Wuhan, China. Due to its 79.6% genetic similarity to the SARS-CoV-1 sequence, it was designated SARS-CoV-2. The disease caused by this virus was termed COVID-19 by the World Health Organization (WHO) [3].

SARS-CoV-2 enters the human body by binding its spike protein (S) to the angiotensin-converting enzyme 2 (ACE2) receptor [4].

ACE2 is a recognized cellular receptor for SARS-CoV-2 that cleaves angiotensin II, thereby counteracting the vasoconstrictive effect of ACE2. Protein S is a viral entry protein of type I that undergoes processing into two segments, namely, S1 and S2. The first segment, S1, attaches to ACE2, while the S2 segment facilitates viral membrane fusion with the target cell [5].

Infections caused by the SARS-CoV-2 virus trigger an inflammatory response that leads to the increased production of specific cytokines, including interleukins 1β, 6, and 8, as well as IFN-γ. Additionally, these infections activate the NF-kB and JAK pathways, which are essential for viral pathogenicity and play a crucial role in patients with severe COVID-19 [6].

Every year, approximately 400,000 instances of cancer are detected in individuals between the ages of 0 and 18, encompassing children and adolescents. The most frequent neoplasms in this population group are leukemias (48%), central nervous system tumors (21%), lymphomas (11%), and solid tumors such as neuroblastoma (6%) and Wilms tumors (5%). Among adolescents (15–19 years of age), the most common types of cancer are central nervous system tumors and lymphomas, leukemias, gonadal cancer (testicular and ovarian), and germ cell tumors. In developed countries, overall survival reaches more than 80%, while in developing countries, it can range from 15% to 45% [7].

The reasons why the weakened immune system in children with cancer increases their vulnerability to COVID-19 remain uncertain, primarily because severe cases and the emergence of complications are infrequent. Therefore, this review gathers information on SARS-CoV-2 treatment that allows physicians to understand the impact of this disease and thus develop strategies to effectively care for pediatric patients with COVID-19 in an oncology context.

## 2. Epidemiology of COVID-19 in Pediatric Cancer Patients

The World Health Organization declared the COVID-19 outbreak a pandemic on 11 March 2020. As of 12 July 2023, 767 million confirmed cases have been registered worldwide, and more than 6.9 million deaths have been recorded. During the week of 5–12 July 2023, 191,922 new cases were reported [8].

While SARS-CoV-2 can infect all children, the majority will experience mild symptoms or show no symptoms at all. Approximately 10% of all COVID-19 cases in the United States are in children. Research findings indicate that children under age 10 are less likely to contract SARS-CoV-2 than individuals over age 20. Nevertheless, if admitted, patients will need intensive care unit (ICU) treatment as often as necessary [9]. Children who have pre-existing conditions, such as obesity, diabetes, asthma, congenital heart disease, genetic disorders, or nervous system conditions, are at a greater risk of experiencing severe illness due to COVID-19 [10].

It remains uncertain which category of children is most prone to experiencing complications. Nevertheless, studies have focused on children with pre-existing conditions, such as chronic lung or heart disease, cancer, severe neurological impairments, immunosuppression, or children who are critically ill [11]. Different studies have shown that cancer patients have an increased susceptibility to contracting COVID-19 due to different factors, such as a compromised immune system due to disease and/or chemotherapy, increased expression of the ACE2 and TMPRSS2 genes, nutritional status, and vulnerability to viral infections [12]. Dai et al., 2020, showed that cancer patients were significantly more susceptible (*p* < 0.01) to in-hospital infections (20 out of 105 patients, 19.04%) than were noncancer patients (8 out of 536 patients, 1.49%) [13].

## 3. Complications in Pediatric Patients with Cancer and COVID-19

**Figure 1:**
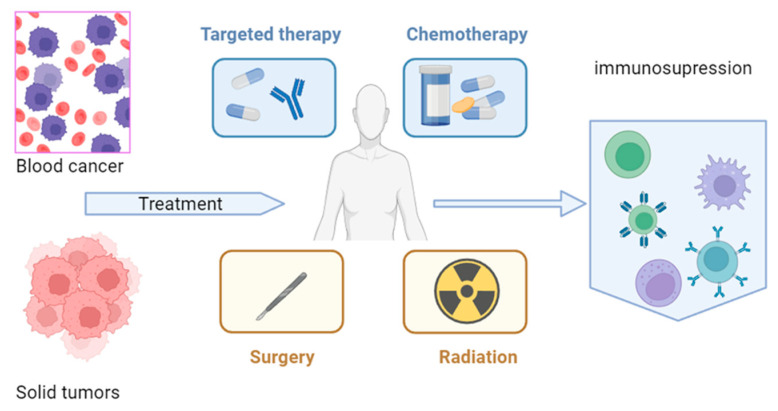
Leukemia, hematologic disease, chemotherapy, and radiation can produce immunosuppression in pediatric patients. Modified with biorender.com (accessed on 12 February 2024).

Cancer is a disease that puts patients in a vulnerable situation by itself or through the treatments used. Leukemia and hematological disease can produce immunosuppression (Figure 1). Usually, this situation worsens in clinical situations. In 2020, the first reports showed that patients with cancer, including children, had greater morbimortality [13]. However, immunosuppression is a protective factor against infection by SARS-CoV-2, and the systemic inflammatory response is responsible for disease severity [14].

The rate of COVID-19-related complications in this group was not greater than that in the general population. This meta-analysis revealed a 4% mortality rate in pediatric patients with hematological malignancies, in contrast to a 34% mortality rate in adults [15]. In the pediatric population with solid and hematologic neoplasms, the need for intensive care was equal to that of pediatric patients, and the mortality rate was 0.6% [14]. With regard to specific complications, thrombosis was one of the principal risk factors for death (28%), and the risk factors for death were age older than 12 years, multisystem inflammatory syndrome, central venous catheter use, and active cancer [16].

It is crucial to keep in mind that these patients exhibit a reduced immune response to the vaccine; therefore, surveillance should continue [17] because viral load is a potential predictor of outcome [18].

Finally, one of the greatest concerns is the consequences of delaying chemotherapy during periods when patients have COVID-19. A total of 58% of the Wecklawek participants had an average delay of 14 days [19].

## 4. Factors Involved in the Response to COVID-19 Treatment in Pediatric Cancer Patients

Clinical results are contingent on various factors, including age, the level of ACE2 expression, and comorbidities. As a result of their advanced age, adult cancer patients face an increased risk of experiencing negative consequences if they contract SARS-CoV-2, have a higher expression of ACE2, and have more comorbidities. Arous et al. reported a greater likelihood of severe COVID-19 in significantly immunosuppressed children, even though the reasons are unclear [20]. Liang et al. reported a series of patients with a greater incidence of intensive care unit admission, invasive ventilation, and mortality than adult patients without cancer (*p* = 0.003) [21]. These findings were also proven by an Italian study that assessed fatality among 355 patients, of whom 20.3% had active neoplasia [22].

Conversely, an investigation was carried out in the United Kingdom from 12 March to 31 July 2020, in which 54 COVID-19 cases were identified among pediatric cancer patients. Among them, 15 (28%) showed no symptoms, 34 (63%) had mild infections, and 5 (10%) experienced moderate, severe, or critical infections. Fortunately, there were no fatalities; only three patients needed ICU support. The estimated rate of hospital-identified SARS-CoV-2 infection in children under 16 years of age with cancer was 3% [23]. The mechanisms through which pediatric cancer patients are more susceptible to SARS-CoV-2 infection are not yet fully known. However, IL-6 has been proposed to be one of the primary disease enhancers, given its role in the tumor microenvironment [24]. A phase II clinical trial is currently in progress to assess the use of tocilizumab in adult patients with COVID-19 and hematopoietic or solid malignant tumors. However, no similar studies involving the pediatric population have been conducted [25].

## 5. COVID-19 Treatment in the Context of Pediatric Cancer Patients

Multiple research groups have proposed various medications for the treatment of COVID-19 based on in vitro tests; these therapies include corticosteroids, antimalarials such as hydroxychloroquine (HCQ) and antivirals (Remdesivir, favipiravir, lopinavir, or ritonavir), convalescent plasma therapy, anti-inflammatory antibodies (imatinib, baricitinib, tocilizumab, tofacitinib, sarilumab), intravenous immunoglobulin, colchicine, ivermectin, nitazoxanide, proxalutamide, and molnupiravir [26]. Different treatments and combinations have been administered to pediatric cancer patients, yielding varying responses, which are detailed in Table 1.

### 5.1. Hydroxychloroquine (HCQ)

Hydroxychloroquine is an FDA-approved medication with lysosomotropic properties, which means that it elevates the pH within lysosomes and disrupts the autophagy/lysosomal degradation process. HCQ can hinder immune function by interfering with antigen processing, antigen presentation, and cytokine production. Additionally, it can suppress the replication of SARS-CoV-2 in laboratory settings. The majority of research indicates that HCQ does not effectively prevent COVID-19 or lower the likelihood of death in hospitalized COVID-19 patients [36]. Hence, it is not advisable to use chloroquine or hydroxychloroquine (HCQ) as standalone treatments or in conjunction with azithromycin for managing COVID-19 in both hospitalized and nonhospitalized individuals, except within the context of clinical trials [37].

### 5.2. Interleukin (IL) Inhibitors

In their serum, COVID-19 patients exhibit elevated levels of proinflammatory cytokines, such as IL-6, IL-1β, IFNγ, IP10, and MCP1 [6]. Patients who need admission to the ICU exhibit the most significant increases in GCSF, IP10, MCP1, MIP1A, and TNFα levels. This indicates that the cytokine storm is linked to the severity of the illness. Trials to block hypercytokinaemia have been conducted worldwide and have demonstrated certain positive clinical outcomes [25]. Treatments under investigation for managing COVID-19 involve IL-6 inhibitors, including antibodies such as tocilizumab and sarilumab. Tocilizumab significantly improves COVID-19 severity in patients with hypercytokinaemia, while sarilumab trials are ongoing [38].

### 5.3. Antiviral Protease Inhibitors

Another approach for treating COVID-19 involves combining the protease inhibitors lopinavir and ritonavir, which are typically used for managing human immunodeficiency virus (HIV) infections [39]. However, a combination of 400 mg and 100 mg of these inhibitors did not offer significant advantages to COVID-19 patients (a total of 199 individuals) compared to conventional single-patient care [40].

Treatment did not alter the clinical progress of patients, did not lower mortality rates, and did not decrease the amount of viral RNA. Nevertheless, when lopinavir–ritonavir were combined with interferon-beta-1b and the antiviral medication ribavirin, the presence of SARS-CoV-2 notably decreased. This combination treatment reduced the average duration from the study’s outset until a negative nasopharyngeal test from 12 days to 7 days, in contrast to lopinavir–ritonavir treatment alone [41]. Nonetheless, certain significant factors could contribute to this inconsistency, such as the tumor type and stage, the tumor’s treatment, the time lapse before admission, the patient’s age, and any coexisting health conditions.

### 5.4. Corticosteroids

In cancer patients, dexamethasone is advised to mitigate inflammation and suppress the body’s immune reaction [42]. A study by Cook et al. revealed that dexamethasone in cancer patients leads to decreased CD4^+^ and CD8^+^ T cell populations while activating immunosuppressive regulatory T cells. These CD4^+^ and CD8^+^ T cell populations are also reduced in COVID-19 patients. Moreover, T cells in COVID-19 patients exhibit significantly elevated levels of PD-1, a marker associated with T cell depletion and cell death. Consequently, the treatment of cancer patients, particularly those receiving cancer therapies, for COVID-19 with drugs that have immunosuppressive effects could increase the risk of severe complications and opportunistic infections [43]. A systematic review released in 2021 noted that this category of medication decreased mortality rates and shortened the duration of mechanical ventilation in patients with acute respiratory distress syndrome caused by COVID-19 [44].

### 5.5. Remdesivir (RDV)

Remdesivir is a prodrug that inhibits RNA-dependent RNA polymerase; inside the cell, it is converted into Remdesivir triphosphate, which becomes part of the RNA transcript synthesized by RNA polymerase and stops the process, preventing further virus replication [45].

This broad-spectrum antiviral agent was initially used to manage Ebola; however, given the need to find a drug to treat the emerging disease of COVID-19, multiple in vitro and in vivo studies and multicenter clinical trials have been conducted.

Finally, in May 2020, the FDA approved Remdesivir as a treatment for emergency COVID-19, and five months later, it was accepted for use in adults and children over 12 years of age weighing at least 40 kg who are sick with COVID-19 and require hospitalization [45,46].

The evidence for the use of Remdesivir in children is limited and becomes even scarcer when the focus is on pediatric patients with cancer. A literature review revealed some case reports with favorable results regarding clinical improvement in pediatric patients with acute lymphoblastic leukemia treated with this antiviral [47,48,49]. However, in cohort studies carried out in children with more heterogeneous cancer diagnoses, despite favorable results, the common conclusion is that more evidence is needed, more studies are required, and more research on the use of Remdesivir in this population is needed [32,33].

### 5.6. Paxlovid^TM^ (Nirmatrelvir + Ritonavir)

Paxlovid^TM^ was the first oral antiviral approved by the FDA to treat COVID-19 in adults; an emergency use authorization was issued on 6 November 2021. It is a drug based on the antiviral nirmatrelvir and the antiretroviral ritonavir. Nirmatrelvir, a hydroxymethylketone derivative, is a peptidomimetic inhibitor of chymotrypsin-like protease (3CLpro or main protease), which is essential for viral replication, integrity, and functionality, and is a highly conserved protease among coronaviruses. An advantage of using this therapeutic target is that no human proteases analogous to 3CLpro have been described [50]. On the other hand, ritonavir, an antiretroviral protease inhibitor used as a pharmacokinetic enhancer, inhibits nirmatrelvir metabolism mediated by the CYP3A enzyme, increasing its antiviral bioavailability.

The emergency authorization also included “qualified” pediatric patients aged 12 to 18. In cohorts in which the efficacy of Paxlovid^TM^ was evaluated in pediatric patients and in patients with oncologic conditions, the viral clearance time was significantly lower in those treated with Paxlovid^TM^ (*p* = 0.033), but the levels of markers of inflammation were significantly greater (*p* < 0.05) [34]. On the other hand, Yan reported in his cohort that he found no significant differences in adverse effects, disease severity, or viral clearance times [35]. These data differ from those observed in studies with adult populations and those that included oncology patients; for example, Weng reported that the duration of hospitalization was significantly shorter in the treated group (*p* = 0.004), the time to viral clearance was also significantly shorter in those treated (*p* = 0.001), and there was a 75.51% reduction in the relative risk of moving from moderate to severe disease [51].

Guermazi et al., 2024 (unpublished data currently under peer review) reported that, in their cohort, the mortality rate was significantly lower in the group receiving Paxlovid^TM^ than in the control group (*p* < 0.001); in addition, patients who did not receive Paxlovid^TM^ or molnupiravir were 1.52 times more likely to have a greater peak need for supplemental oxygen than those treated with these drugs [52]. Few studies have been conducted in the pediatric oncology population. Therefore, their efficacy remains controversial, and their use could be implemented on a cost-benefit basis, considering that certain benefits have been demonstrated in adults with oncologic conditions. Furthermore, as in adults, it should be noted that the FDA contraindicates this drug for patients using tyrosine kinase inhibitors since ritonavir increases plasma concentrations and has adverse side effects.

## 6. Use of Cancer Drugs in Clinical Trials for COVID-19 Treatment

There are certain similarities in symptoms between cancer patients and COVID-19 patients. As a result, utilizing anticancer medications for COVID-19 treatment might benefit patients [53]. Cancer medications are safe and efficacious and are capable of addressing inflammation, immune issues, and the replication of viruses. Table 2 lists the anticancer drugs currently being explored for the treatment of COVID-19.

There are other drugs, such as monoclonal antibodies of target therapies used to treat cancer, that could have some ability to treat patients with COVID-19, including ruxolitinib, bevacizumab, nivolumab, and pembrolizumab. However, more evidence of their effects still needs to be provided [61].

## 7. Implementation of Chemotherapy Application Rooms in Children with COVID-19

In more than 60% of pediatric cancer patients who tested positive for COVID-19, there was an observed delay in their chemotherapy, with a median delay of 28 days. This situation is alarming, as it decreases dose intensity and increases the risk of relapse and death [62]. As we better understood how COVID-19 impacts children and adolescents with cancer, it became necessary to evaluate the pros and cons of continuing chemotherapy even when they tested positive for COVID-19. The introduction of segregated rooms specifically for administering chemotherapy to children with ongoing infections was initiated. Among 77 children receiving active systemic treatment, 70 (approximately 90.9%) received intravenous chemotherapy without experiencing severe toxic side effects [63].

## 8. Considerations Regarding Pediatric Cancer Patient Care in the COVID-19 Era

The research conducted by Bouland and colleagues proposed that pediatric cancer patients may not be at greater risk than other children with regard to becoming infected with SARS-CoV-2 [64] or experiencing associated health problems; nevertheless, it is essential to ensure that the care provided to pediatric cancer patients adheres to social distancing guidelines and the proper use of personal protective equipment.

Conversely, potential interactions between the chemotherapeutic drugs and medications used for treating COVID-19 can pose challenges. Several hospitals have restricted elective surgeries for solid tumors to minimize the risk of COVID-19 transmission. Only essential surgical procedures vital for the survival of cancer patients are permitted [65].

Another noted issue is the decline in patients receiving medical care. A multicenter study in Middle Eastern countries conducted in 29 pediatric hospital centers showed a decrease of up to 45% in the number of patients treated [66]. The pandemic had both positive and negative impacts. On the positive side, it led to the development of mRNA-based vaccines, an increase in the use of telemedicine, the emergence of new technologies, and the creation of new education platforms. However, on the negative side, the pandemic also resulted in increased mortality rates, domestic violence, increased unemployment rates, increased poverty, and increased biomedical waste [67].

This phenomenon increased telemedicine consultations, a mechanism adopted in countries such as Norway (health care by telephone/video call) to prevent any delay in diagnosis and treatment [68].

Furthermore, the COVID-19 pandemic has led to unparalleled disruption in the realm of research, resulting in the closure of numerous laboratories and a notable deceleration in the progress of clinical trials [69]. Many scientists and physicians are changing their research lines to study the impact of SARS-CoV-2 on cancer patients [70].

Despite these precautions, providing the recommended treatment for each patient remains essential based on the specific type of tumor and its stage [71]. Regarding this issue, the research conducted by Capozza and colleagues offers additional confirmation of the significance of delivering regular cancer care to children and young individuals with malignancies, irrespective of the challenges posed by the COVID-19 pandemic. It also offers a blueprint for potential cancer patient registries [72].

## 9. Coinfections in Pediatric Patients with COVID-19

The immunosuppressed state of oncology patients treated with chemotherapy increases their susceptibility to infections [13]. Although COVID-19 infection in children is usually uncomplicated, the fragile state of children with cancer may increase the impact of SARS-CoV-2 or favor a coinfection that complicates disease progression. A study by Escobar et al. (2021), which included 201 children with cancer infected with SARS-CoV-2, reported that confirmed coinfections correlated with an increased risk of severe disease (OR = 1.95) and death (OR = 5.2) [73]. In another study of 131 patients diagnosed with cancer or hematopoietic stem cell transplantation who were PCR-positive for SARS-CoV-2, bacterial coinfections (*Escherichia coli*, *Staphylococcus aureus*, and *Mycobacterium tuberculosis*) were observed in seven patients, five patients had viral coinfections (cytomegalovirus, enterovirus/rotavirus, parainfluenza, and herpes simplex virus), three patients had invasive fungal infections (*Aspergillus* spp., *Pneumocystis jirovecii*, and *Candida tropicalis*), and coinfections were significantly associated with increased disease severity (1.74; 95% CI: 1.03–3.03) [74].

Gotzinger et al. (2020) detected additional viruses in respiratory specimens, including enterovirus, rhinovirus, influenza, parainfluenza, adenovirus, respiratory syncytial virus, bocavirus, coronavirus NL63, coronavirus HKU1, coronavirus OC43, and human metapneumovirus, in oncology patients with COVID-19. Coinfection increased the likelihood of having signs or symptoms of upper or lower respiratory tract infection compared to infection with SARS-CoV-2 alone and significantly increased the likelihood of ICU admission, ventilator support, and inotropic support [75].

Other relevant features have been observed in pediatric oncology patients with SARS-CoV-2. For example, Corso (2021) reported a mortality of 12.3%, in contrast to the 1% reported for the general pediatric population [76]. This difference may be due to various parameters, such as tumor stage IV (*p* = 0.029), intubation (*p* < 0.001), thrombocytopenia < 25,000 cells/mm^3^ (*p* < 0.001), D-dimer > 1 µg/mL (*p* = 0.003), clinical malnutrition (*p* = 0.0023) [76,77], and disseminated intravascular coagulation (*p* = 0.03) [77]. Another factor influencing mortality was recent chemotherapy (OR 2.09 CI 95% 1.09–4.08; *p* = 0.028) [78]. In addition, the type of tumor influences the course of infection; for example, patients with hematological malignancies have a greater risk of COVID-19 infection than patients with solid tumors and present a more critical clinical picture requiring invasive ventilation (OR 1.57, 95% CI 1.15–2.15, *p* < 0.0043) [78].

## 10. Conclusions

The outlook for pediatric cancer patients who contract SARS-CoV-2 is favorable compared to that for adults. Most patients will be asymptomatic or have mild disease; only 2–3% will require admission to a hospital ICU for ventilatory support. Consequently, altering or postponing the cancer treatment regimen may not be needed, particularly for patients who exhibit no symptoms or who only experience mild symptoms of viral disease. Since there is no specific treatment for the pediatric oncological population with COVID-19, self-medication is discouraged to avoid overdose and adverse effects. Hence, conducting in-depth research is essential for creating therapeutic compounds to combat this global pandemic. Individuals with pre-existing health conditions, including cancer, are at greater risk of experiencing severe outcomes due to COVID-19 than the general population. Therefore, pediatric cancer patients should exercise increased caution, and hospitals must establish robust management strategies to minimize the negative impact of the COVID-19 pandemic on vulnerable cancer patient groups. SARS-CoV-2 is a novel infection that has emerged globally, and even with advancements in medical science, we anticipate the occurrence of many more cross-species infections in the future.

COVID-19 represents a significant challenge in pediatric cancer patients. Vaccines, like in the rest of the population, were beneficial for these patients. New strategies, such as the use of chemotherapy, have been implemented for children with cancer who are positive for COVID-19 but asymptomatic since the risk of disease progression is greater than the risk of complications from SARS-CoV-2.

## Figures and Tables

**Table 1:** Treatments for COVID-19 in pediatric cancer patients.

Study	Age (Years)	N	Oncology Diagnoses	Treatment for COVID-19	Outcome/Chemotherapy Status	Levels of Evidence *
Montoya, 2021 [26]	6 (0.8–16)	69	Acute lymphoblastic leukemia (ALL; *n* = 36), non-Hodgkin lymphoma (*n* = 5), brain tumor (*n* = 5), Wilms tumor (*n* = 4), acute myeloid leukemia (AML; *n* = 3), bone tumor (*n* = 3), soft tissue tumor (*n* = 3), and other (*n* = 12)	Ivermectin, azithromycin, and corticosteroids (*n* = 9); any treatment (*n* = 60)	In total, 62 patients are alive with no complications, and 7 died. Chemotherapy was suspended in all cases.	LEVEL 4
De Rojas, 2020 [27]	10.6 (0.6–18)	15	Hematological malignancies (*n* = 11) and solid tumors (*n* = 4)	HCQ (*n* = 8); a combination of azithromycin, tocilizumab, lopinavir-ritonavir, corticoids, and Remdesivir (*n* = 3); any treatment (*n* = 4)	Favorable clinical outcomes: hospital stay was 8 days. Chemotherapy had to be interrupted or delayed in six patients (40%).	LEVEL 4
Wang, 2020 [28]	5.1 (0.1–16)	255	Leukemia (*n* = 160), solid tumors (*n* = 31), aplastic anemia (*n* = 21), lymphoma (*n* = 18), Langerhans cell histiocytosis (*n* = 14), hemophagocytic syndrome (*n* = 6), and myelodysplastic syndrome (*n* = 5)	Continue chemotherapy regimen and supplementary oxygen	Only 4 patients needed ICU admission. NCD.	LEVEL 3
Mehrvar, 2021 [29]	9.17 ± 1.5	17	ALL (*n* = 4), AML (*n* = 3), brain tumor (*n* = 5), lymphoma (*n* = 3), rhabdomyosarcoma (*n* = 1), and osteosarcoma (*n* = 1)	Oseltamivir, HCQ, and azithromycin	In total, 14 patients showed clinical improvement, and 3 patients died despite treatment. Cancer-directed therapies were suspended for all patients until recovery from COVID-19 symptoms, except for two patients who had finalized their treatment and were receiving follow-up examinations.	LEVEL 3
Saultier, 2022 [30]	8	1	ALL	Anti-SARSCoV-2 mAbs (bamlanivimab and etesevimab)	mAbs are tolerated undergoing therapy for leukemia. Chemotherapy was given along with antibodies.	LEVEL 3
Parker, 2022 [18]	12 (0–24)	87	ALL (*n* = 53), AML (*n* = 2), Hodgkin (*n* = 2), NHL (*n* = 2), solid tumors (*n* = 22), and other (*n* = 6)	Remdesivir (*n* = 14), Steroids (*n* = 11), Monoclonal Ab (*n* = 2), Convalescent plasma (*n* = 4), HCQ (*n* = 1), Azithromycin (*n* = 2), and Other (*n* = 1)	There were no COVID-19-related deaths; two patients died from progression of cancer. Twenty-six patients required hospitalization for management of COVID-19 manifestations. Four patients needed multiple hospitalizations due to recurrent fever and respiratory symptoms. Among the 64 patients who were in the midst of anticancer therapy, 22 (34%) experienced modifications or delay in anticancer therapy (median delay = 14 days). Anticancer therapy was delayed or modified in 13 of 31 patients and 9 of 33 patients in the midst of intensive and non-intensive anticancer therapy, respectively. Among the eight patients who presented with COVID-19 at the time of cancer diagnosis, chemotherapy was delayed or modified up to 2 weeks in two (both with acute leukemia), and there was no worsening of COVID-19 with intensive chemotherapy in the seven patients who required an intensive regimen.	LEVEL 2+
Tolunay, 2022 [31]	8.6 (4.3–13.5)	45	Leukemia/lymphoma (*n* = 41), solid tumors (*n* = 4)	Antibiotic therapy (*n* = 45), favipiravir (*n* = 8), steroid (*n* = 8), and HCQ (*n* = 1)	Multiorgan failure and death (*n* = 2), severe COVID-19 disease (*n* = 4). Chemotherapy or radiotherapy was interrupted due to COVID-19.	LEVEL 3
Hammad, 2021 [32]	9 (1–18)	76	ALL/LL (*n* = 38), AML (*n* = 20), lymphoma (*n* = 5), CML (*n* = 3), neuroblastoma (*n* = 3), RMS/NRMS (*n* = 3), CNS tumors (*n* = 3), and others (*n* = 1)	Remdesivir (*n* = 45), no Remdesivir (*n* = 31)	Favorable overall survival in both groups; one patient developed a 2-fold elevation in serum creatinine above baseline as an adverse effect to Remdesivir. Chemotherapy was delayed in 59.2% of patients with a median of 21 days (5–71 days). Of these, 42.5% were delayed for ≤2 weeks, and doses were modified in 30.2%.	LEVEL 2−
Ling, 2023 [33]	6.5 (1–18)	18	Lymphoma, ALL, AML, post-hematopoietic stem cell transplant recipients, juvenile myelomonocytic leukemia, optic glioma, atypical teratoid/rhabdoid tumor (ATRT), and pilomyxoid astrocytoma	Remdesivir at a loading dose of 5 mg/kg/dose (maximum 200 mg) on day one and 2.5 mg/kg/dose every 24 h (maximum 100 mg) on days two and three (*n* = 4). No Remdesivir (*n* = 14)	Remdesivir treatment was safe but did not lead to early clearance of SARS-CoV-2 or a shorter time to negative PCR. There was no need to delay chemotherapy in patients treated with Remdesivir.	LEVEL 2−
Li, 2023 [34]	4–14	20	Acute lymphoblastic leukemia (*n* = 14) and chronic myelogenous leukemia (*n* = 1)	Paxlovid^TM^ (Group A) (adult dose) (body weight)/40 kg <20 kg once a day for 5 days >20 kg twice a day for 5 days (*n* = 6) Adult dose: nirmatrelvir 300 mg/ritonavir 100 mg Group B (non-Paxlovid^TM^ group) (*n*= 9)	Clinical symptoms were similar in both AB groups. Group A: the fever lasted 1–6 days, and clinical symptoms resolved in 1–30 days. Virus Clearance Time 6.44 days. CRP (mg/L) 15.60. PCT (ng/mL) 0.447 Group B: the fever lasted 0–3 days, and clinical symptoms resolved for 2–14 days. Virus Clearance Time 15.20 days. CRP (mg/L) 0.447. PCT (ng/mL) < 0.05 The virus clearance time in group A was shorter than in group B (*p* = 0.033) The CRP and PCT inflammatory indexes in group A were significantly higher than in group B (*p* < 0.05). NCD	LEVEL 2++
Yan, 2022 [35]	6–14	35	Leukemia (*n* = 1)	Paxlovid^TM^ (*n* = 5) For children aged 12–14 years with body weight >40 kg, nirmatrelvir/ritonavir 300 mg/100 mg orally twice daily for 5 days. For children aged 6–14 years with body weight 20–40 kg, 150 mg/100 mg twice daily for 5 days. For children aged 6–14 years with body weight <20 kg, 150 mg/100 mg once daily for 5 days. No Paxlovid (*n* = 30)	One patient treated with PaxlovidTM developed diarrhea, and one had elevated liver enzymes. The respective viral shedding times were 11, 4, 10, 9, and 9 days. All patients were discharged to their homes without abnormal findings on the second-week follow-ups. Not treated with Paxlovid^TM^: there were no differences in symptom spectrum, disease severity, incidences of diarrhea, elevated liver enzymes, and viral shedding times. NCD.	LEVEL 2++

ALL: acute lymphoblastic leukemia; AML: acute myeloid leukemia; ICU: intensive care unit; NCD: no chemotherapy data; * Scottish Intercollegiate Guidelines Network (SIGN) classification was used to evaluate levels of evidence, available at https://www.sign.ac.uk/media/2039/sign50_2015.pdf (accessed on 16 April 2024).

**Table 2:** Anticancer drugs used for the treatment of COVID-19.

Anticancer Drug	Use in Childhood Cancer	Clinical Trials
Methotrexate	Acute lymphoblastic leukemia, brain tumors, non-Hodgkin lymphoma, and osteosarcoma	[54]
Tretinoin	Acute promyelocytic leukemia	[55]
Dexamethasone	Leukemia, lymphoma, and brain tumors	[44,56]
Etoposide	Leukemia, brain tumors, germ cell tumors, retinoblastoma, lymphoma, neuroblastoma, Ewing sarcoma, Wilms tumors	[57]
Imatinib	Acute lymphoblastic leukemia	[58]
Prednisone	Acute and chronic lymphoblastic leukemia and lymphoma	[59]
Interleukin-2	Neuroblastoma and melanoma	[60]
